# Transcriptome Analysis of the Intracellular Facultative Pathogen *Piscirickettsia salmonis*: Expression of Putative Groups of Genes Associated with Virulence and Iron Metabolism

**DOI:** 10.1371/journal.pone.0168855

**Published:** 2016-12-29

**Authors:** Alvaro Machuca, Victor Martinez

**Affiliations:** FAVET-INBIOGEN, Faculty of Veterinary Sciences, University of Chile, Santiago, Chile; Texas A&M University Health Sciences Center, UNITED STATES

## Abstract

The intracellular facultative bacteria *Piscirickettsia salmonis* is one of the most important pathogens of the Chilean aquaculture. However, there is a lack of information regarding the whole genomic transcriptional response according to different extracellular environments. We used next generation sequencing (NGS) of RNA (RNA-seq) to study the whole transcriptome of an isolate of *P*. *salmonis* (FAVET-INBIOGEN) using a cell line culture and a modified cell-free liquid medium, with or without iron supplementation. This was done in order to obtain information about the factors there are involved in virulence and iron acquisition. First, the isolate was grown in the Sf21 cell line; then, the bacteria were cultured into a cell-free liquid medium supplemented or not with iron. We identified in the transcriptome, genes associated with type IV secretion systems, genes related to flagellar structure assembly, several proteases and sigma factors, and genes related to the development of drug resistance. Additionally, we identified for the first time several iron-metabolism associated genes including at least two iron uptake pathways (ferrous iron and ferric iron uptake) that are actually expressed in the different conditions analyzed. We further describe putative genes that are related with the use and storage of iron in the bacteria, which have not been previously described. Several sets of genes related to virulence were expressed in both the cell line and cell-free culture media (for example those related to flagellar structure; such as basal body, MS-ring, C-ring, proximal and distal rod, and filament), which may play roles in other basic processes rather than been restricted to virulence.

## Introduction

In Chile, Piscirickettsiosis is the most important bacterial disease in salmonid fish and causes high mortalities and significant economic losses during the sea water phase (approximately U$ 100 million per year) (reviewed in [1]). The etiologic agent is *P*. *salmonis*, a facultative intracellular Gram-negative bacterium [2], phylogenetically related to *Francisella*, *Coxiella*, and *Legionella* [3]. The bacterium is a non-motile coccoid organism with an approximate diameter of 0.5–1.5 μm that is often a pleomorphic, fastidious, and facultative intracellular pathogen in host cells. It has been seen replicating inside intracytoplasmic vacuoles in macrophages of head kidney of infected salmon. *In vitro* can be cultured in cell lines as CHSE-214 (Chinook salmon embryo), macrophage/monocyte (RTS-11) or Sf21 (Spodoptera frugiperda; ECACC 89070101) cell culture, where *P*. *salmonis* has high titers) [4]; and also in cell-free culture media, as CHAB agar, AUSTRAL-SRS broth or MC medium [5–7].

The virulence levels of *P*. *salmonis* have been described using mortality curves in different salmonid species [8–11]. Several genes have been used to assess virulence based on the available *P*. *salmonis* genome. The presence of a locus encoding a toxin-antitoxin system (TA) was detected and named *ps-tox-antox*. Expression of the *ps-tox* gene was highly toxic in transformed *Escherichia coli*, showing minimal growth after 8 hours compared to those cells carrying the expression vector with both, the *ps-antox* and *ps-tox* genes, which showed a normal growth curve [12]. Using a stressful cell-free culture media (Marine broth, Difco), Marshall et al [13] described that *P*. *salmonis* produced a large amount of cellular aggregates that form a typical biofilm structure that act as an adaptive strategy for survival and persistence under stress conditions in the marine environment. Using RT-qPCR, the expression of an operon associated with biofilm formation (*mazEF*) was demonstrated, reaching its highest levels of expression during the early stage of formation of this structure [13]. In addition, the in vitro expression of bacterial genes in the RT11 cell line show that four virulence genes were expressed (*dotA*, *dotB*, *icmK* and *icmE*; that are homologous to the type IV secretion system Dot/Icm) in *P*. *salmonis*. [14]. A recent experiment using the SHK-1 line, suggested that *P*. *salmonis* significantly increased the gene expression of two homologues of the *clpB* and *bipA*, when compared with a cell free media [15]. These genes show increased expression allowing the pathogen to evade the cellular immune response and replicate inside macrophages [15].

The study of the transcriptome is essential for the functional interpretation of many molecular components of cells that are related to iron metabolism and virulence, thus improving the knowledge about disease pathogenesis [16]. The transcriptome of a cell corresponds to the complete set of transcripts for a specific developmental stage or under a particular physiological condition [17]. Currently, next generation sequencing (NGS) of RNA (RNA-seq) has emerged as a powerful tool for gene expression studies that allows the discovery of novel genes that have not been previously annotated and the mapping of transcriptional structures of a variety of bacterial species [18]. RNA-seq has been used to characterize *in vitro* differences at the whole transcriptome level between field strains of *Salmonella* enteritidis with high and low pathogenicity where the latter showed a unique transcriptional profile characterized by a significant reduction in the expression of several transcriptional regulators, genes involved in virulence mechanimsm and in the protection against osmotic and oxidative stress [19]. RNA-seq has also been used to study the transcriptomic response in other phylogenetically related bacteria, such as *Legionella pneumophila* [20,21]. *Mycobacterium marinum* [22], which is another pathogen causing disease in fish, show a similar mechanism of persistency like *P*. *salmonis*.

The iron metabolism pathways are known in several Gram negative bacteria, but this information is lacking in *P*. *salmonis*. For example the transcriptomic analysis of *Pseudomonas fluorescens* Pf using RNAseq revealed that genes encoding functions related to iron homeostasis, a number of TonB-dependent receptor systems, and some inner-membrane transporters were significantly up-regulated in response to iron limitation. Conversely, the expression of genes involved in hydrogen cyanide production and flagellar biosynthesis were down-regulated under these conditions [23]. The transcriptional response of the Gram negative bacterium *Campylobacter jejuni* was also characterized using RNA-seq. The results identified 77 iron-activated genes and 50 iron-repressed genes that were involved in iron acquisition or oxidative stress defense, revealing that iron is an important regulator of flagellar biogenesis [24]. This demonstrate the usefulness of this approach for discovery new metabolic pathways in prokaryotes.

In this paper, we are aimed at gain knowledge on the pathways related to several mechanisms used for iron metabolism and virulence in *P*. *salmonis*. We present for the first time information concerning the transcriptomic response of *P*. *salmonis* via NGS technology and the pathways involved in the growth of this bacterium in different culture conditions. In particular, considering media lacking iron, which is expected to be an important factor involved in bacterial growth [25]. We perform a series of experiments using different cultures (a cell line and a liquid medium supplemented or not with iron) to describe the full transcriptomic response of *P*. *salmonis* under these conditions.

## Materials and Methods

### Bacterial strain and experimental culture conditions

In order to develop a highly proliferative intracellular environment, a cryopreserved isolate of *P*. *salmonis* (FAVET-INBIOGEN) was propagated in the Sf21 cell line (*Spodoptera frugiperda*; ECACC 89070101) [4,26] in antibiotic-free Grace’s medium supplemented with 10% fetal calf serum. Infected monolayers were incubated at 26°C in 25-cm^2^ culture flasks. Bacterial cells were harvested when the infected monolayers reached 95–100% cytopathic effects (CE). A supplemented liquid medium modified from Henriquez et al. [5] was used that contained 8 g L^-1^ yeast extract, 4 g L^-1^peptone from meat, 1.32 g L^-1^ (NH_4_)_2_ SO_4_, 0.1 g L^-1^ MgSO_4_, 6.3 g L^-1^ K_2_HPO_4_, 9 g L^-1^ NaCl, 0.08 g L^-1^ CaCl_2_, 3% glucose and 0.03 g L^-1^ FeSO_4_.

### Experimental design

Sf21 cells from 4 culture flasks containing monolayers infected with *P*. *salmonis* that reached 95–100% CE were centrifuged. Then, an aliquot of 2 ml from the supernatant named “CC medium” (cell culture medium) was centrifuged at 4000 x g for 10 min at 4°C. The pellet was used for total RNA extraction. The rest of the supernatant was also centrifuged, and the pellet was cultured in liquid medium supplemented or not with FeSO_4_ named “CFC-I medium” (cell-free culture medium with iron supplementation) and “CFC-N medium” (cell-free culture medium without iron supplementation), respectively. The infected media were incubated at 25°C and 100 rpm. After 24 h, a pool of bacterial pelleted from each condition (CFC-I and CFC-N), was obtained for total RNA extraction.

### RNA isolation procedures

Total RNA was extracted using TRIzol (Invitrogen) following the manufacturer’s instructions. The RNA pellets dissolved in diethylpyrocarbonate (DEPC)-treated water were quantified using the Qubit^®^ fluorometer (Invitrogen) and stored at -80°C. The quality of RNA extraction was analyzed according to the RNA quality number (RQN) using the Fragment Analyzer^®^.

### Library preparation and transcriptome sequencing

To sequence the transcriptome, RNA samples were treated with DNase I (Fermentas) and purified with the RNeasy MinElute Cleanup Kit (Qiagen) to remove traces of DNase I. Depletion of rRNA was accomplished using the Ribo-Zero^™^ Kit (Bacteria)–Low Input. Purification of the rRNA-depleted samples was accomplished with the RNeasy MinElute Cleanup Kit (Qiagen). RNA-seq libraries of the samples were prepared using the ScriptSeq^™^ v2 RNA-Seq Library Preparation Kit (Epicentre^®^) according to the manufacturer’s protocol. The assessment of the library quantity and quality were completed using qPCR (Illumina) and the Fragment Analyzer^®^, respectively. The samples were sequenced using the v2 Reagent Kit (300 cycles PE kit) with NGS equipment (Illumina Miseq^®^).

### Mapping of reads and description of transcriptomes

The reads obtained from the CFC and CC media samples were mapped to the annotated genome of the FAVET-INBIOGEN (Whole Genome Shotgun (WGS) project accession number LELB01000000) isolate using the CLC Genomics Workbench. The *P*. *salmonis* transcriptomes were characterized according to the functional classification of the Rast server [27] and Kyoto Encyclopedia of Genes and Genomes (KEEG) through the automatic metabolic pathway annotation server KAAS [28]. According to Humphrys et al [29], we defined two different cut-offs to asses a minimal gene expression following sequencing. The minimal gene expression was considering for a gene with a minimum of a RPKM (Reads Per Kilobase of transcript per Million mapped reads) ≥ 0.1 and a minimum of 10 mapped reads. Additionally, to assess the relative abundance of genes between both grown conditions, we used the test of proportion through Kal’s test statistic using CLC Genomic Workbench [30], The genes were considered as differentially expressed with FDR (false discovery rate) ≤ 0.01.

## Results

### Comparisons between transcriptomes obtained under different culture conditions

*P*. *salmonis* from the CC medium had 2188 genes with a minimal level expression (RPKM ≥ 0.1 and a minimum of 10 mapped reads). In the CFC-I medium, *P*. *salmonis* expressed 2264 genes and 2296 in the CFC-N medium with a minimal level expression.

We validate some of the genes annotated in the RNA-seq using a RT-qPCR. The chosen genes were ferrous iron transport protein B (*feoB*), ferric uptake regulation protein (*fur*), bacterioferritin (*bfr*), Ferrichrome transport ATP-binding protein (*fhuC*), RNA polymerase sigma factor RpoS (*rpoS*) and Ferrochelatase, protoheme ferro-lyase (*hemH*).

**Fig 1:**
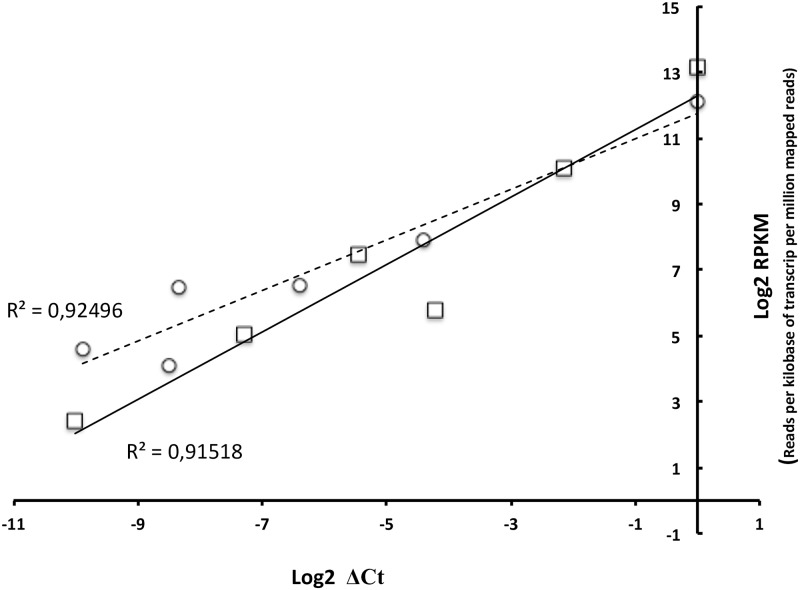
Correlation between RT-qPCR and RNA-seq data for six genes (*feoB*, *fur*, *bfr*, *fhuC*, *rpoS* and *hemH*) expressed by *P*. *salmonis* in both types of cultures. The tendency line and determination coefficient (R^2^) are showed for both cultures.

For the 6 genes used, there was a high correlation between Ct and RPKM (Read per kilobase per million mapped reads) values for each gene in both samples (Fig 1).

A total of 1205 genes were differentially expressed, where 876 genes were up regulated and 328 genes were down regulated in CFC-I culture medium compare to CC culture medium with FDR ≤ 0.01. The number of differentially expressed genes related to virulence is presented in Table 1. When we compared CFC-I with CFC-N, this analysis revealed 323 genes differentially expressed (FDR ≤ 0.01), with 145 genes up regulated when iron was supplemented in the media. A description of the number of genes related to virulence, differentially expressed presented in Table 2.

**Table 1:** Number of genes up and down regulated in the transcriptome from CFC medium compare to CC medium.

	Up regulated genes	Down regulated genes
**Flagellar structures**	1	3
**Iron metabolism**	23	7
**Proteases**	6	10
**Drug resistance**	8	8
**Oxidative stress**	20	6
**T4SS**	0	8
**Chaperones**	5	3
**Sigma factors**	6	1

**Table 2:** Number of genes up and down regulated in the transcriptome from CFC-I medium compare to CFC-N medium.

	Up regulated genes	Down regulated genes
**Flagellar structures**	0	0
**Iron metabolism**	5	4
**Proteases**	1	2
**Drug resistance**	5	2
**Oxidative stress**	3	4
**T4SS**	2	0
**Chaperones**	1	1
**Sigma factors**	1	1

### Pathways associated with virulence and iron metabolism

We sought to divide the analysis in two main mechanisms that the bacteria could use to interact with the host. In particular, we analyzed virulence factors such as flagellar structure, type IV secretion system, sigma factors, proteases, chaperones and mobile proteins elements, as described previously. We also analyzed the pathways related with uptake, utilization and storage of the iron (see below).

### Virulence-associated genes

#### Flagellar structures

**Fig 2:**
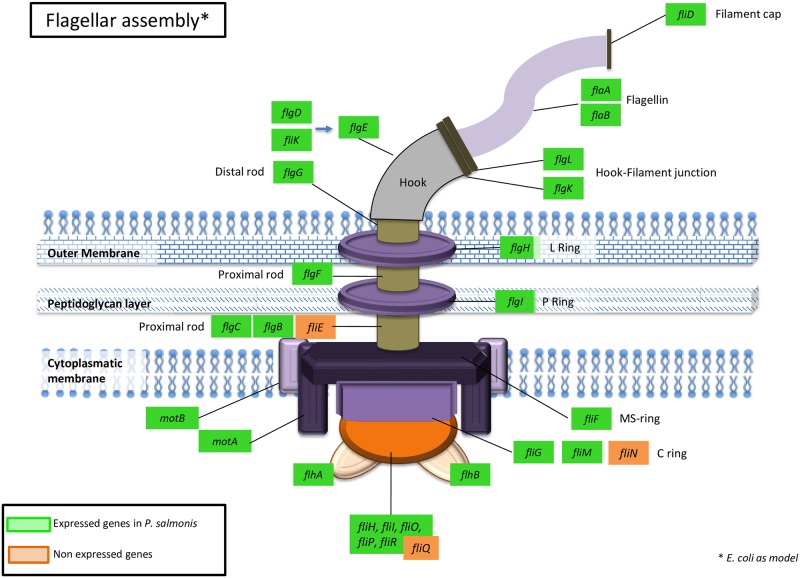
Representative genes associated with the flagella in the transcriptome. The image shows the set of genes associated with the flagellar structure that are expressed by *P*. *salmonis* in all conditions tested. The green square represents genes expressed in the different media, whereas the orange square represents genes that did not show expression., in the different grown conditions analyzed.

Although this pathogen has been described as a non-motile bacterium, a total of 46 genes involved in flagellum biosynthesis, assembly, structural organization, function, and the flagella motor genes were expressed in *P*. *salmonis* under different conditions (Fig 2). Among these, three genes were differentially expressed in CC medium when it was compared with the CFC-I medium: *flagellar motor rotation protein MotB_2*, *Flagellar hook-associated protein FlgL* and *Chemotaxis regulator-transmits chemoreceptor signals to flagelllar motor components CheY_3*, which were 4.7, 1.9 and 1.6 fold more expressed in CC medium (FDR ≤ 0.01) (S1 Table). When we compared CFC-I with CFC-N, we did not find any genes differential expressed between treatments.

All these flagella related genes have been involved in protein export (especially of virulence factors) in non-motile bacteria. Several proteins that form the flagellar metabolic pathway may export other proteins to the extracellular space, especially those linked to virulence [31,32]. Maezawa et al. [33] reported the existence of hundreds of flagellar-expressed hooks and basal body structures in a bacterium that lacked the filament portion of the flagellum, supporting previous suggestions of the possible specialization of these genes in protein export from the bacterium to the host. Abby and Rocha [34] suggested that non-flagellar type III secretion systems (injectisomes) evolved from the bacterial flagellum. Additionally, *P*. *salmonis* can produce extracellular products (EPCs), at least some of which are thermo-labile exotoxins that probably play a role in the pathogenesis of Piscirickettsiosis [35]. Overall, the results showed that *P*. *salmonis* expressed several genes that were associated with the flagellar structure; However, the *fliC* gene that encodes the filament of the flagellum was not found in the annotated genome. Moreover, the subunits *flaA* and *flaB* which also encode the filament of the flagella were expressed. This results show that *P*. *salmonis* is expressing another host-cell adaptation system (i.e., the type III secretion system), which likely delivery virulence factors such as flagellar component and exotoxins into eukaryotic cells. Further analysis of this secretion system are required in order to understand the interaction of these factors in the host.

#### The type IV secretion system

Data from our experiment showed that the FAVET-INBIOGEN isolate expressed 24 genes annotated in the *icm*/*dot* system. Among this group, eight genes were differentially expressed in CC medium (FDR ≤ 0.01) compared with CFC-I (S1 Table), and two were differentially expressed between CFC-I and CFC-N (S2 Table). The group of genes that form the *icm* (intracellular multiplication)/*dot* (defective organelle trafficking) system have been described as a type IV secretion system (T4SS) that is responsible for intracellular survival and multiplication. T4SS has been described in *L*. *pneumophila* and *C*. *burnetii* as one of the most important virulence factors of these pathogens [36–39], which are both pathogens, are phylogenetically related to *P*. *salmonis*.

#### Proteases

Bacterial pathogens express a group of proteases that contribute to virulence either directly due to host interactions or indirectly (e.g., allowing replication in the host environment) [40]. Because *P*. *salmonis* is a facultative intracellular bacterium, we would expect to find expression of alternative proteases related to these processes that have not been previously described in this bacterium. Indeed, the transcriptome of the FAVET-INBIOGEN isolate revealed 20 genes (e.g., *clpP*, *clpA*, *clpS* and *clpX*) encoding proteases and chaperons that were expressed under all culture conditions and would contribute to bacterial adaptation to the hostile conditions produced by host cells [41]. Moreover, *clpB*, whose expression has been described previously in *P*. *salmonis*, was differentially expressed in the CC medium compared with CFC-I medium (FDR ≤ 0.01) (S1 Table), but it was not differentially expressed between CFC-N and CFC-I.

#### Sigma factors

Bacterial pathogens have alternative sigma factors that regulate the expression of virulence genes and virulence-associated genes in response to a particular stimuli, which can be critical for host establishment [42]. Alternative sigma factors are a class of proteins that constitute essential dissociable subunits of the prokaryotic RNA polymerase [43]. The association of appropriate alternative sigma factors with the core RNA polymerase provides a mechanism for cellular responses mediated through the redirection of transcription initiation [44].

The transcriptomic responses of the FAVET-INBIOGEN isolate under all culture conditions showed 10 genes related with sigma factors. Seven were differentially expressed between CC medium and CFC-I (S1 Table), and two between CFC-I and CFC-N (S2 Table). Among those expressed genes; *rpoE*, encoding the sigma factor E (σ^E^) that controls specific functions involved in the interaction of the bacterium with the host (e.g., synthesis, assembly and homeostasis of lipopolysaccharides and outer membrane porins including several proteases) [45]; *rpoD*, which has been described as a housekeeping gene in Gram-negative bacteria; *rpoH*, which is associated with the heat-shock response; *rpoS*, which contributes to bacterial survival in stressful environments [44]; and *rpoN*, which has been reported to be involved in flagellar regulation [46].

#### Chaperones

Molecular chaperones are essential for the survival of all three kingdoms of life and have been shown to play key roles in the integration of cellular and organismal responses to environmental fluctuation. Heat shock proteins (Hsp) comprise a subgroup of molecular chaperones that are induced in response to adverse environmental conditions. These proteins have been reported for several bacteria during infection processes, and thus form a first line of attack that helps consolidate pathogen virulence [47]. Similar to many other pathogenic bacteria, we expect that *P*. *salmonis* would express several groups of chaperones that were important for the invasion process in the host. Indeed, numerous genes encoding Hsp33, Hsp40, Hsp60, Hsp70 and Hsp90 family proteins were annotated in the transcriptomes of *P*. *salmonis*. The genes encoding the heat shock protein GrpE, GroES, GroEL, Hsp90 and Hsp70, which were more expressed in the CC medium, and the gene encoding the Hsp33, hsp40 and “Protein export cytoplasm chaperone” proteins were more expressed in the CFC-I medium (FDR ≤ 0.01) (S1 Table). When we compared CFC-I with CFC-N only 2 genes were not consistently differentially expressed, *hsp40* which was more expressed in CFC-I and *hsp90* which was more expressed in CFC-N (FDR ≤ 0.01) (S2 Table).

#### Mobile protein elements

The analysis of the transcriptomes in this study revealed more than forty annotations defined as "mobile protein elements", which are defined as DNA segments encoding enzymes and other proteins that allow movement of the genomic DNA [48]. Sixteen of them were differentially expressed between CC medium and CFC-I (S1 Table), and seven between CFC with and without iron supplementation (S2 Table) (FDR ≤ 0.01).

### Iron metabolism-associated genes

As mentioned before, few studies are available in the literature regarding iron metabolism in *P*. *salmonis*. Thus, to characterize the mechanisms underlying iron homeostasis in this bacterium is important, in order to understand more deeply how the bacteria interact with the host. In all the media used, a total of 41 genes related to iron homeostasis were expressed and sorted into four functional groups: uptake/transport, intracellular utilization, storage and.iron regulation (S1–S3 Figs).

#### Iron uptake

The transcriptomic response of *P*. *salmonis* was associated with three pathways involved with the transport for siderophores. Both transcriptomes contained homologues of vibrioferrin (*pvsA*, *pvsB*, *pvsC*, *pvsD*, *pvsE*, and *pvuA*), a member of the carboxylate class of siderophores that has been described in marine a bacteria [49,50]. Among them, *pvsB*, *pvsC*, *pvsD*, *pvsE* were differentially expressed in CC medium versus the CFC-I medium (FDR ≤ 0.01) (S1 Table). Additionally, a homologue of *fhuC*, which is involved in the iron uptake by siderophores of the hydroxamate class, was also represented in both transcriptomes, but more expressed in CC medium (FDR ≤ 0.01) (S1 Table). A homologue of the *feoB* gene and other of the *feoA* gene that are take up Fe^2+^ directly were expressed in both cultures media. In addition, a homologous gene *fecD* that is involved in iron uptake by siderophores of the dicitrate class was also contained in all transcriptomes analyzed of the bacteria (associated with CC, CFC-I, CFC-N), but without showing statistical differences between both grown conditions.

When we compared CFC-I with CFC-N two genes were differentially expressed (*feoA* and *pvuA*) which were more expressed in CFC-N (FDR ≤ 0.01) (S2 Table).

#### Iron utilization

Iron-sulphur [Fe-S] clusters and ferredoxins (*fdx*) play an important role in cellular processes, such as bacterial respiration, cofactor biosynthesis and gene expression [51]. As expected, the [Fe-S] cluster assembly pathway was represented in both growth conditions assayed for *P*. *salmonis* by genes encoding for the following proteins: a cysteine desulphurase that catalyzes the release of sulphur from L-cysteine, which is one of the essential components of the CFC medium; the SufE protein that accepts the sulphur molecule that is necessary for [Fe-S] cluster assembly [52]; an ATPase SufC protein and two assembly proteins (SufB and SufD) [53]; a gene encoding a likely iron binding protein from the SufA family; and a gene associated with the iron binding protein IscA, which is involved in [Fe-S] cluster assembly. All these genes were over represented in CFC-I medium in relation to CC medium (FDR ≤ 0.01) (S1 Table). More importantly, we detected the expression of *iscR*, which is a necessary regulator of [Fe-S] cluster assembly [54,55] and that was 6-fold more represented in CFC-I medium than CC medium (FDR ≤ 0.01) (S1 Table).

Comparing CFC-I with CFC-N there were four genes differentially expressed (*pvuA*, *4Fe-4S ferredoxin-iron sulfur binding*, *IscR* and *probable iron binding protein from the HesB_IscA_SufA family*) (FDR ≤ 0.01), which three of them more expressed in CFC-I (S2 Table).

#### Iron storage

As expected, all the transcriptomes contained the *bfr* gene that encodes bacterioferritin, which was 1.4 fold more expressed in CFC-I medium than CC medium (FDR ≤ 0.01) (S1 Table), and 1.06 fold more expressed in CFC-I than CFC-N medium (S2 Table). This protein is involved in intracellular iron storage and has been described as the most important storage protein for iron in bacteria [56,57].

#### Iron regulation

Similar to many other prokaryotes, iron homeostasis in *P*. *salmonis* appear to be regulated by the *fur* gene. The *fur* gene that encodes the main transcriptional regulator of iron genes [58,59] was almost 3-fold more represented in CFC-I medium when compared with the CC medium (FDR ≤ 0.01) (S1 Table). However, the “*RNA-binding protein hfq*” gene was also differentially expressed in all comparison (S1 and S2 Tables). This gene has been described to encode a chaperone of a small RNA named RhyB that was also responsible for iron homeostasis, albeit at a different level than *fur* [60,61]. A further analysis of the small RNA will be required in order to understand more deeply this mechanism.

#### Oxidative stress

High concentrations of iron result in an oxidant environment that catalyzes reactive species of oxygen, mainly through the Fenton reaction. These molecules are toxic causing cell death [62,63]. Several expressed genes were observed under both conditions, e.g: alkyl hydroperoxidase D (*ahpD*), alkyl hydroperoxide reductase C (*ahpC*), catalase (*kat*) and superoxide dismutase (*sodB*), which are associated with protection of the cell against oxidative stress and were up regulated in the CFC-I medium compared with CC medium (FDR ≤ 0,01) (S1 Table). Other seven genes were differentially expressed between CFC-I and CFC-N, four of them were more expressed in CFC with iron supplementation (FDR ≤ 0.01) (S2 Table).

## Discussion

### Genes associated with virulence processes

Our results showed that 46 genes were associated with flagellar biosynthesis, assembly, structural organization, and function (S3 Table). To date, *P*. *salmonis* has been described as a non-motile bacterium [1]. However, in this type of facultative intracellular symbiotic bacteria the presence of the flagella is unnecessary and energetically expensive unless proteins involved in the flagella pathway are also involved in other essential functions for the bacterium or the host [31].

During *in vivo* infection, *P*. *salmonis* replicates in the phagocyte preventing phagosome fusion with the lysosome. Therefore, the bacterium requires the expression of the necessary metabolic machinery to survive under these conditions [1,64,65] The expression of the Dot/Icm type IV secretion system (*icmK*, *icmE*, *dotA* and *dotB*) was previously described in *P*. *salmonis* [14]. Our results demonstrated that those *dot/icm* gene homologues were expressed both during *in vitro* cell line infection and in cell-free media, suggesting putative constitutive expression, confirming thereby the hypothesis previously suggested by Gomez et al. [14]. In *L*. *pneumophila*, this Type IV secretion system shows cytopathogenicity and hemolytic properties in addition to being essential for the induction of apoptosis [66,67]. To understand and analyze the functions of proteins delivered into host cells by this mechanism, it is important to improve the knowledge of the mechanisms related with *P*. *salmonis* pathogenesis.

The stressful conditions of the phagosome (i.e., oxidative burst and fusion of the phagosome with lysosomes) are capable of inducing the expression of bacterial chaperones (heat shock proteins, Hsp) and proteases, which also participate in the survival strategies of intracellular pathogens. Our results suggest similar survival strategies due to the expression of genes that are homologues of the most important classes of Hsps described in bacteria (Hps33, DnaJ, DnaK, HtpG, GroEL, GroES and GrpE proteins). All these proteins are involved in protein folding processes in *E*. *coli*, *B*. *suis*, *C*. *jejuni* and *S*. *enterica* serovar Typhimurium [47,68–70]. At present, DnaK protein is the best characterized bacterial chaperone. Mutations in *dnaK* increase the sensitivity of normally resistant *S*. *aureus* strains to oxacillin and methicillin; moreover, mutations in *dnaK* or *dnaJ* in *E*. *coli* make the cells susceptible to fluoroquinolones [71].

The FAVET-INBIOGEN isolate also expressed the *clpP* and *clpB* genes that encoded ATP-dependent proteases that were described as important virulence factors in numerous bacterial pathogens [72]. The represented *clpA* and *clpX* genes in the transcriptomes have been described in Gram-negative bacteria [40]. These genes encode two ATPase-chaperones that interact with the ClpP protease [41]. In *L*. *pneumophila*, the ClpP homologue is required for cell division and several transmission traits, including stress tolerance, cell shortening, sodium sensitivity, cytotoxicity and intracellular multiplication [73]. Moreover, the ClpB protease contributes to resistance to various stress conditions, intracellular multiplication and virulence in *F*. *tularensis* [74]. The presence of the *clpB* gene has been described in the *P*. *salmonis* showing significantly increased expression during infection of the SHK-1 cell line compared to the cell-free medium, indicating that *clpB* could also be expressed during propagation in host cells permitting the adaptation to the hostile conditions in the host [15]. We found similar results in our study, i.e. the *clpB* gene was significantly more expressed in the CC medium (Sf21 cell line), thereby confirming the hypothesis of Isla et al. [15]. Despite the fact that phenotypic protein expressions have not yet have been demonstrated in *P*. *salmonis*, data concerning its presence in the genome and its gene expression will allow the study of alternative mechanisms of survival in the host.

### Metabolic pathways and iron uptake

Previously, four putative genes encoding siderophore-related proteins involved in iron metabolism and transport (one *hemH* gene, two *tonB* genes, and one *fur* gene) were reported in the published genome of *P*. *salmonis* [75]. The results of the transcriptome analysis of our isolate revealed two pathways for iron uptake: through siderophores and iron ferrous uptake.

Several Gram-negative bacteria synthesize and excrete low-molecular-weight compounds that chelate iron with high affinity that are called siderophores [76]. Siderophores are synthesized and secreted for iron acquisition; for example, muscle tissues expressing carrier proteins in *Atlantic salmon* were recently demonstrated to have a significantly higher bacterial load and became a niche where *P*. *salmonis* could acquire iron [25]. The FAVET-INBIOGEN isolate expressed genes encoding a protein system homologous to a system present in *V*. *parahaemolyticus* that was involved in the biosynthesis of vibrioferrin [77]. Because *P*. *salmonis* is a Gram-negative bacterium, the expressed genes also encoded proteins of the TonB system, which is a sophisticated mechanism that supplies the required energy for ferri-siderophores uptake. The TonB system includes an integral membrane protein (ExbB), a membrane-anchored periplasmatic protein (ExbD) and the TonB protein [78,79].

The *feo* system is the main route described for the uptake of Fe^+2^ and is associated with the growth of bacteria under anaerobic or microaerobic [80,81] and to a lesser extent aerobic conditions. This system was described in *E*. *coli* K-12; however, it has also been described in *Salmonella* [82], *Campylobacter* [83], *Francisella* [84] and *Legionella* [85]. The expression of *feoB* serves as an alternative pathway for iron uptake by *P*. *salmonis*.

The RNA-seq data show that bacterioferritin (*bfr*) is a mechanism that the bacteria is using for iron storage. This is a ubiquitous system used for the other facultative intracellular bacteria, such as *Salmonella* and *B*. *abortus* [86,87]. When intracellular iron concentrations are very high, this family of proteins also plays an antioxidant role and sequesters iron away from oxygen free radicals [88].

To assembly the [Fe-S] cluster (in addition to serving as a source of iron), the bacterium requires a source of sulphur. The sulphur is supplied by L-cysteine, an amino acid with a thiol side chain, which is an important supplemental component of the CFC medium. After transport into the cytoplasm, the cysteine desulphurase catalyzes the release of sulphur from L-cysteine, which is necessary for [Fe-S] cluster assembly [89]. Cysteine supplementation is not required for intracellular growth in macrophages, mammalian cell lines, or natural amoebic hosts, indicating that cysteine and other essential amino acids are obtained directly from the host [90].

An environment with excess iron together with reactive oxygen species (ROS) via the Fenton reaction causes oxidative stress. This stress increases the damage associated with [Fe–S] clusters, protein carbonylation, Cys/Met-residue oxidation, membrane lipid peroxidation, and DNA damage. The main strategy employed is the production of enzymes that degrade ROS species to maintain stress levels within a range of tolerance [91]. Our bacteria expressed alkyl hydroperoxidase D (*ahpD*) and alkyl hydroperoxide reductase C (*ahpC*), which provide significant antioxidant protection and have been described in various bacteria [92–94]. Additionally, Chung et al. [95] demonstrated that deletion of the *ahpC* genes alone in *V*. *parahaemolyticus* did not significantly affect the survival of this pathogen; thus, bacteria require more than one of these antioxidant molecules. Resistance against oxidative stress through biofilm formation was studied in *C*. *jejuni*, where the over expression of *ahpC* was associated with a decrease in biofilm formation; conversely, an *ahpC* mutant increased biofilm formation [96].

Finally, one of the most important factors involved in the development of resistance to antibiotics is the ability of bacteria to share genetic resources through lateral gene transfer (LGT) [97]. Drug resistance genes are commonly associated with mobile genetic elements (the mobilome) that can be transferred between bacteria by plasmids, integrons and transposons [98].

### Final remarks

Several sets of virulence-associated genes were expressed under the experimental conditions studied here; however, the response of the bacteria was similar in different media. Virulence-associated genes were expressed in the cell line culture and also in the cell-free medium supplemented with iron, indicating that these genes could play a role in basic processes rather than specifically in pathogenesis. Furthermore, the expressed genes related to flagellar structure could play a similar role to a type III secretion system during pathogenic processes, supporting the production of extracellular products by *P*. *salmonis* [35], but still this require further analysis considering the interaction with the host.

Overall, this study provides new insights into the functional mechanisms of *P*. *salmonis*. This information will be important for improving our basic knowledge of this bacterium to gain better prevention and control strategies against Piscirickettsiosis in Chile.

## Supporting Information

S1 Table Differentially expressed genes related with virulence and iron metabolism between CC medium and CFC-I medium. (XLSX)

S2 Table Differentially expressed genes related with virulence and iron metabolism between CFC-I medium and CFC-N medium. (XLSX)

S3 Table Total expressed genes in the three grown conditions. (XLSX)

S1 Fig Genetic organization and hypothetical model of siderophores in *P*. *salmonis*. (A) Suggested genetic organization of siderophore synthesis and transport genes transcribed from the *P*. *salmonis* genome that could represent a putative operon. The RPKM values of each of genes that were expressed in the pathway are showed for the CFC-I growth condition. (B) Siderophore synthesis is accomplished by the PvsA, PvsB, PvsD and PvsE proteins. The PvsC protein transports the siderophore to the extracellular space, where it captures Fe^+3^ and is uptaked by the outer membrane protein PvuA. (OM: outer membrane; CM: cytoplasmatic membrane).
(TIFF)

S2 Fig Genetic organization and hypothetical model of genes involved in the transport of siderophores in *P*. *salmonis*. (A) The organization of *tonB*, *ferric aerobactin*, *ABC transporter* and the *iron (III) dicitrate transport system permease protein FecD* genes could represent an operon separated from the *exbD/tolQ* and *motA/tolQ/exbB* proton channel family protein genes. The RPKM values of each of genes that were expressed in the pathway are showed for the CFC-I growth condition. (B) There are two putative genes encoding transmembrane cytoplamatic proteins which transport ferri-siderophores from the periplasmic space into the cytoplasm where these molecules are reduced. (OM: outer membrane; CM: cytoplasmatic membrane).
(TIFF)

S3 Fig Hypothetical genetic organization and assembly model of genes related with [Fe-S] clusters in *P*. *salmonis*. The RPKM values of each of genes that were expressed in the pathway are showed for the CFC-I growth condition. When the cell has high levels of [Fe-S] clusters, the IscR protein would capture a cluster in its structure, which is a signal for transcriptional repression of *sufB*, *sufC*, *sufD*, and *sufS*.
(TIFF)
