# Supplementary figures and images for: Transcriptome Analysis of the Intracellular Facultative Pathogen Piscirickettsia salmonis: Expression of Putative Groups of Genes Associated with Virulence and Iron Metabolism
Source: PLoS One. 2016 Dec 29;11(12):e0168855. doi: 10.1371/journal.pone.0168855 (PMC5199080; doi:10.1371/journal.pone.0168855)

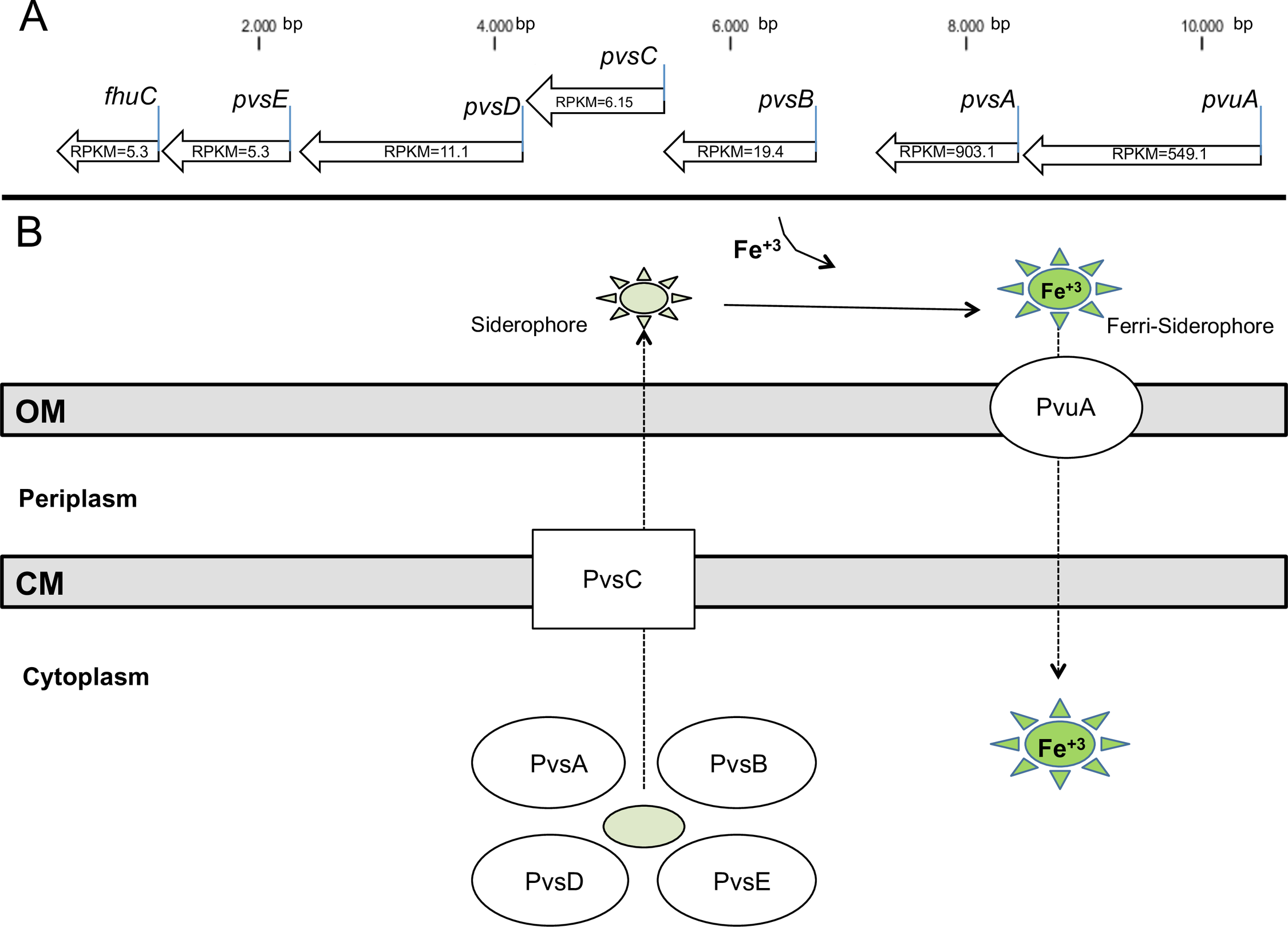

Supplement: S1 Fig — (A) Suggested genetic organization of siderophore synthesis and transport genes transcribed from the P. salmonis genome that could represent a putative operon. The RPKM values of each of genes that were expressed in the pathway are showed for the CFC-I growth condition. (B) Siderophore synthesis is accomplished by the PvsA, PvsB, PvsD and PvsE proteins. The PvsC protein transports the siderophore to the extracellular space, where it captures Fe+3 and is uptaked by the outer membrane protein PvuA. (OM: outer membrane; CM: cytoplasmatic membrane). (TIFF) [file pone.0168855.s004.tiff]

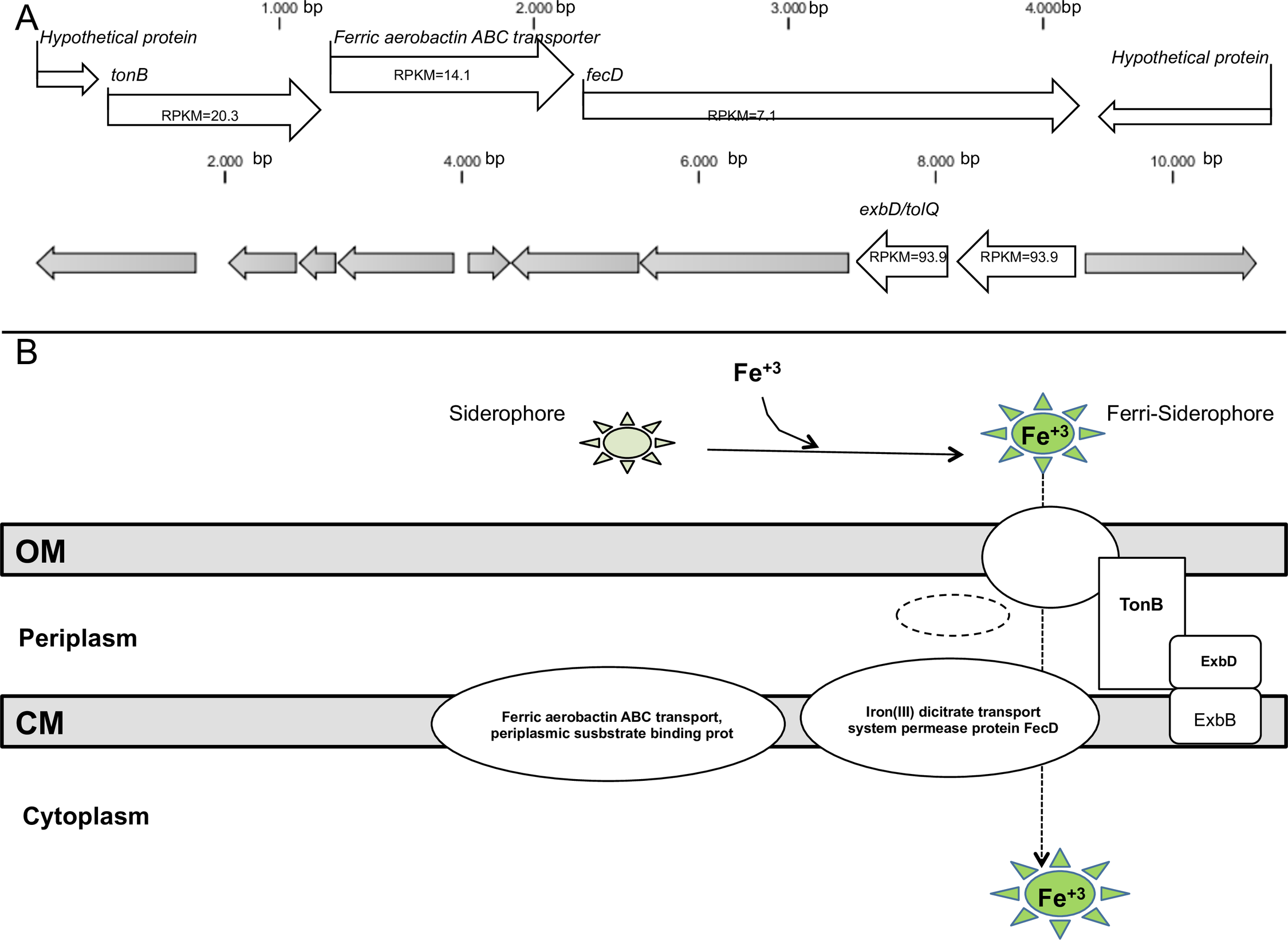

Supplement: S2 Fig — (A) The organization of tonB, ferric aerobactin, ABC transporter and the iron (III) dicitrate transport system permease protein FecD genes could represent an operon separated from the exbD/tolQ and motA/tolQ/exbB proton channel family protein genes. The RPKM values of each of genes that were expressed in the pathway are showed for the CFC-I growth condition. (B) There are two putative genes encoding transmembrane cytoplamatic proteins which transport ferri-siderophores from the periplasmic space into the cytoplasm where these molecules are reduced. (OM: outer membrane; CM: cytoplasmatic membrane). (TIFF) [file pone.0168855.s005.tiff]

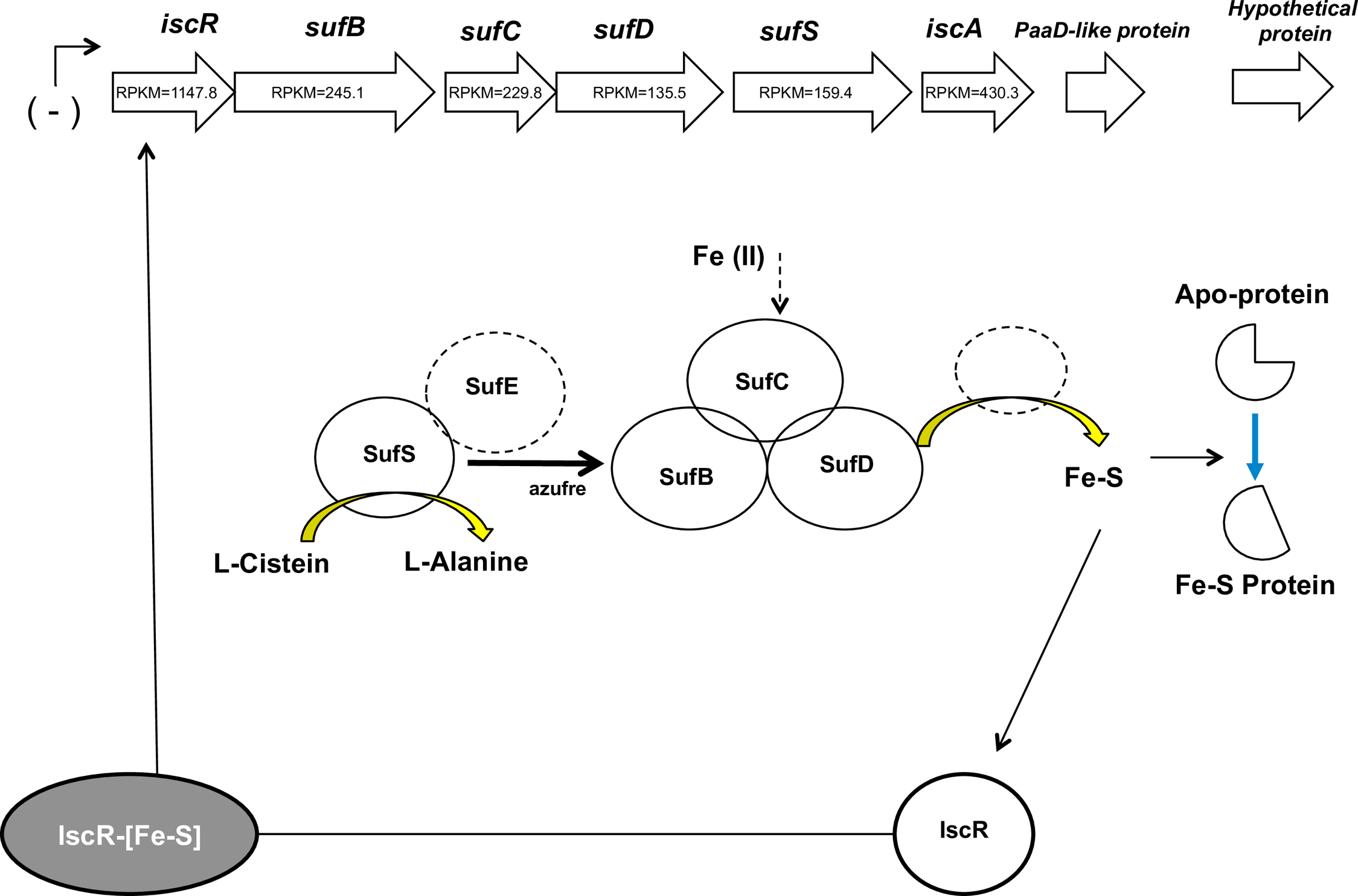

Supplement: S3 Fig — The RPKM values of each of genes that were expressed in the pathway are showed for the CFC-I growth condition. When the cell has high levels of [Fe-S] clusters, the IscR protein would capture a cluster in its structure, which is a signal for transcriptional repression of sufB, sufC, sufD, and sufS. (TIFF) [file pone.0168855.s006.tiff]
